# Selective Peroxisome Proliferator-Activated Receptor Alpha Modulators (SPPARMα) in the Metabolic Syndrome: Is Pemafibrate Light at the End of the Tunnel?

**DOI:** 10.1007/s11883-020-00897-x

**Published:** 2021-01-03

**Authors:** Jean-Charles Fruchart, Michel P. Hermans, Jamila Fruchart-Najib, Tatsuhiko Kodama

**Affiliations:** 1 Residual Risk Reduction Initiative (R3i) Foundation, Picassoplatz 8, 4010 Basel, Switzerland; 2 Division of Endocrinology and Nutrition, Cliniques Universitaires St-Luc and Institut de Recherche Expérimentale et Clinique (IREC), Université Catholique de Louvain, Brussels, Belgium; 3 Laboratory for System Biology and Medicine. Research Center for Advanced Science and Technology, The University of Tokyo, Tokyo, Japan

**Keywords:** Pemafibrate, Selective peroxisome proliferator-activated receptor alpha modulator, SPPARM, Triglycerides, Metabolic syndrome, Non-alcoholic fatty liver disease

## Abstract

**Purpose of Review:**

Adoption of poor lifestyles (inactivity and energy-dense diets) has driven the worldwide increase in the metabolic syndrome, type 2 diabetes mellitus and non-alcoholic steatohepatitis (NASH). Of the defining features of the metabolic syndrome, an atherogenic dyslipidaemia characterised by elevated triglycerides (TG) and low plasma concentration of high-density lipoprotein cholesterol is a major driver of risk for atherosclerotic cardiovascular disease. Beyond lifestyle intervention and statins, targeting the nuclear receptor peroxisome proliferator-activated receptor alpha (PPARα) is a therapeutic option. However, current PPARα agonists (fibrates) have limitations, including safety issues and the lack of definitive evidence for cardiovascular benefit. Modulating the ligand structure to enhance binding at the PPARα receptor, with the aim of maximising beneficial effects and minimising adverse effects, underlies the SPPARMα concept.

**Recent Findings:**

This review discusses the history of SPPARM development, latterly focusing on evidence for the first licensed SPPARMα, pemafibrate. Evidence from animal models of hypertriglyceridaemia or NASH, as well as clinical trials in patients with atherogenic dyslipidaemia, are overviewed.

**Summary:**

The available data set the scene for therapeutic application of SPPARMα in the metabolic syndrome, and possibly, NASH. The outstanding question, which has so far eluded fibrates in the setting of current evidence-based therapy including statins, is whether treatment with pemafibrate significantly reduces cardiovascular events in patients with atherogenic dyslipidaemia. The PROMINENT study in patients with type 2 diabetes mellitus and this dyslipidaemia is critical to evaluating this.

## Introduction

The metabolic syndrome poses a global challenge as societies become increasingly urbanised, sedentary and obese. A key requirement for identification is the combination of three or more of the following: increased waist circumference, elevated triglycerides (TG), low plasma concentration of high-density lipoprotein cholesterol (HDL-C), elevated blood pressure and raised fasting blood glucose (Table 1) [1]. Worldwide, 20–30% of adults are affected, although this varies with age, ethnicity and gender [2]. The metabolic syndrome is no longer a disease of affluence, with escalating prevalence in emerging regions coincident with rising rates of obesity [3, 4], or a disease of adults, as globally more than 5% of children and adolescents are affected [4, 5]. The extensive comorbidity of the metabolic syndrome confers a substantial burden, from the increased risk for atherosclerotic cardiovascular disease (ASCVD), affecting multiple vascular territories [6], and type 2 diabetes mellitus [7, 8]. In fact, ASCVD and diabetes are the two leading causes of death.

**Table 1:** Harmonised definition of the metabolic syndrome. Derived from Alberti et al [1]

Waist >94 cm (men) or > 80 cm (women)* together with the presence of two or more of the following:	
Fasting blood glucose greater than 5.6 mmol/L (100 mg/dL) or diagnosed diabetes	
HDL cholesterol < 1.0 mmol/L (40 mg/dL) in men, < 1.3 mmol/L (50 mg/dL) in women or drug treatment for low HDL-C	
Fasting blood triglycerides > 1.7 mmol/L (150 mg/dL) or drug treatment for elevated triglycerides	
Blood pressure > 130/85 mmHg or drug treatment for hypertension	

*Based on the International Diabetes Federation thresholds for Europid population, with subsequent regional-specific definitions in men and women

Beyond the increased risk for ASCVD, metabolic derangements that characterise the metabolic syndrome predispose to the development of non-alcoholic fatty liver disease (NAFLD), the most common chronic liver disease worldwide. NAFLD encompasses the spectrum of disease ranging from asymptomatic fatty infiltration of hepatocytes in the absence of inflammation to progression to non-alcoholic steatohepatitis (NASH), liver fibrosis and liver failure. Among NAFLD patients, about half exhibit the metabolic syndrome with dyslipidaemia the most prevalent characteristic (in ~ 70% of patients) [9]. The pathogenesis of NAFLD is multifactorial, involving genetic, environmental and metabolic factors. Of the latter, TG accumulation in the liver, reflecting the imbalance between free fatty acid influx and efflux/catabolism, is a hallmark feature which also drives inflammation [10, 11]. Globally, it is estimated that NAFLD affects about one billion people, with an overall prevalence of ~ 25% in adults, with the highest rates in South America, the Middle East and Asia [9]. The clinical, economic and health-related quality-of-life burden of NAFLD is already substantial and growing [9].

Thus, given the changing landscape of cardiovascular risk associated with escalating obesity, the metabolic syndrome poses a global socioeconomic challenge. Renewed thinking about therapeutic options is imperative.

## Pathogenesis

**Fig. 1:**
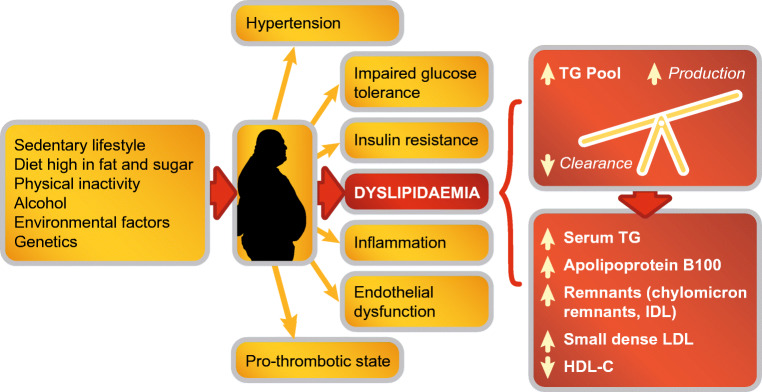
Dyslipidaemia is an important feature of the metabolic syndrome. Overproduction of large very low-density lipoprotein particles is a fundamental defect contributing to the increase in the triglyceride pool. This initiates a sequence of lipoprotein changes, leading to higher levels of remnant particles, an increase in small, dense low-density lipoprotein particles and lower plasma concentration of high-density lipoprotein cholesterol. IDL, intermediate-density lipoprotein; HDL-C, high-density lipoprotein cholesterol; LDL, low-density lipoprotein; TG, triglyceride

A key driver of the metabolic syndrome is visceral obesity, a marker of ectopic fat deposition [12•, 13•] (Fig. 1). Expansion of visceral adipose tissue to a greater extent than that of subcutaneous adipose tissue is associated with metabolic alterations which promote inflammation. Key amongst these metabolic derangements is atherogenic dyslipidaemia, in particular increases in TG-rich lipoproteins, their remnants and apolipoprotein (apo) C-III [14, 15]. These effects are mediated via crosstalk between a multitude of pathways that promote impaired adipogenesis, adipokine dysregulation and inflammation, and increase free fatty acids, oxidative stress, adipose tissue hypoxia and lipotoxicity (both local and systemic) [16••]. Recently, attention has focused on angiopoietin-like protein 2 (ANGPTL2), a glycoprotein which is expressed abundantly in adipose tissue. Under normal conditions, ANGPTL2-mediated expression contributes to angiogenesis and tissue damage repair, whereas overexpression promotes chronic inflammation [17–19]. In obese women with insulin resistance, ANGPTL2 production by adipocytes was shown to upregulate proinflammatory cytokine production in macrophages, in turn increasing adipose tissue inflammation, systemic insulin resistance and hyperinsulinaemia [20]. Thus, ANGPTL2 provides a link between the metabolic syndrome, NAFLD and ASCVD.

Hypertriglyceridaemia is a key component of the dyslipidaemia associated with the metabolic syndrome and NAFLD, its hepatic manifestation. Moderately elevated TG levels (a surrogate for elevated TG-rich lipoproteins and their remnants) result from increased dietary-derived apo B48-containing intestinal chylomicrons, overproduction of hepatic very low-density lipoproteins (VLDL) and reduction in catabolism of TG-rich lipoproteins. Evidence from epidemiologic, mechanistic and genetic studies supports a causal association between TG-rich lipoproteins and their TG-hydrolysed remnants and ASCVD [21•, 22]. Specifically, it is the cholesterol contained within these lipoproteins (i.e. remnant cholesterol) that promotes the development of atherosclerosis and ischaemic heart disease, in part mediated via low-grade inflammation [23, 24]. Postprandial hypertriglyceridaemia is also an emerging contributing factor in residual cardiovascular risk [25•]. Clinically, the combination of elevated TG and increased waist circumference (hypertriglyceridaemic waist) represents a marker of high-risk carotid atherosclerosis features, and highlighting this dual anomaly can improve the identification of individuals with metabolic syndrome and preclinical atherosclerosis beyond traditional risk factors [26•].

## PPAR: a Key Therapeutic Target

Lifestyle intervention, encompassing both dietary changes and increased physical activity, is an important first step in the management of mild to moderate hypertriglyceridaemia, but long-term adherence is usually problematic [27]. Beyond lifestyle, agents that target the nuclear receptor peroxisome proliferator-activated receptor (PPAR) are obvious therapeutic candidates given their role in regulating the expression of key genes involved in adipogenesis, lipoprotein metabolism, inflammation and metabolic homeostasis [28••, 29, 30]. Three isoforms are identified to date, PPARα, PPARγ and PPARβ/δ, each encoded by separate genes [31]. PPARα is abundant in energy-demanding tissues, such as the liver, kidney, heart and skeletal muscle; PPARγ is predominantly found in adipose tissue, macrophages and the large intestine, whereas PPARβ/δ is more ubiquitous in distribution [32, 33]. These PPARs are controlled through their interaction with fatty acids and their derivatives and are the pharmacological targets for the lipid-lowering fibrates (PPARα) or the insulin sensitizer thiazolidinediones (PPARγ).

### PPARα

Activation of PPARα by binding of endogenous ligands (e.g. fatty acids or eicosanoids), or drugs (fibrates) to the ligand binding domain and subsequent heterodimerisation with the ligand-activated retinoid X-receptor (RXR) trigger a conformational change which influences cofactor recruitment, either promoting (transactivation) or inhibiting (transrepression) expression of target genes. This process is mediated by the interaction between the activated PPARα, the PPAR response element (PPRE) of the target gene and relevant cofactors which render the complex transcriptionally active (or inactive in the case of transrepression) [32]. PPARα activation targets key genes involved in TG metabolism, specifically increasing the production of lipoprotein lipase and apo A-V and decreasing plasma levels of apo C-III; increasing HDL synthesis by targeting genes encoding apo A-I and A-II, scavenger receptor BI, and the ATP binding cassette transporters A1 and G1; and enhancing beta-oxidation by increasing expression of hepatic acyl CoA synthase [34–38]. The net effects are reduction in serum TG, an increase in HDL-C concentration, attenuation of very-low-density lipoprotein (VLDL) particles, as well as a shift in the low-density lipoprotein (LDL) profile to fewer small, dense LDL particles and a proportional increase in larger, less dense LDL particles. There is also transrepression of proinflammatory genes, leading to lower levels of inflammatory mediators such as C-reactive protein, interleukin-6 and prostaglandins [39]. Emerging evidence also suggests that PPARα favourably influences glucose homeostasis and insulin sensitivity, possibly mediated via effects on acetyl-CoA [40], and inhibits thrombogenesis and improves vascular function, although the underlying mechanisms are not fully defined.

### Other PPAR Isoforms

PPARγ appears to be important in cell differentiation and energy metabolism, binding to the PPRE of almost all adipogenic genes, including those implicated in glucose and fatty acid metabolism. Although less well characterised, PPARβ/δ appears to regulate lipid metabolism, glucose homeostasis and inflammation, suggesting a role in the maintenance of energy homeostasis [29, 41].

## Current Therapeutic Options

### PPARα

Given their pharmacological profile, PPARα ligands (fibrates) are appropriate treatments for correcting atherogenic dyslipidaemia that is characteristic of the metabolic syndrome. Current fibrates are either specific for PPARα (fenofibrate and gemfibrozil) or activate all three PPAR subtypes (pan-agonist, bezafibrate). While the TG-lowering efficacy of fibrates is well established, their clinical benefit in terms of reduction in cardiovascular events is less convincing. Two prospective trials with gemfibrozil, the Helsinki Heart Study, a primary prevention trial in men with elevated non-HDL-C [42], and the Veterans Affairs High-Density Lipoprotein Cholesterol Intervention Trial (VA-HIT), a secondary prevention trial in men with low HDL-C [43], showed significant reduction in cardiovascular events. It must be borne in mind, however, that both were essentially monotherapy lipid-lowering trials as these were conducted before widespread statin use. Of the remaining trials, two with fenofibrate, Fenofibrate Intervention and Event Lowering in Diabetes (FIELD) [44] and the Action to Control Cardiovascular Risk in Diabetes (ACCORD) Lipid trial [45], both in patients with type 2 diabetes, and one with bezafibrate (Bezafibrate Infarction Prevention [BIP] study) in patients with established coronary disease [46] were inconclusive. None was positive in terms of reduction in their primary endpoint for the total study population, and in the case of the FIELD study, was further complicated by discrepancies in the uptake of statin use between the two groups. The ACCORD Lipid study was the only trial conducted against background statin treatment; the lack of benefit may largely relate to inappropriate patient selection in terms of baseline TG levels and prevalence of atherogenic dyslipidaemia at inclusion [45].

There are, however, important insights from subgroup analyse of the fibrate trials. Analyses of the Helsinki Heart Study and the VA-HIT showed greater reduction in cardiovascular events in patients with the combination of both elevated TG and low HDL-C [47], or with insulin resistance [48], respectively. In the FIELD study, post hoc analysis showed that patients who satisfied the metabolic syndrome criteria (about 80% of the total study population), derived greater clinical benefit, with maximum reduction in cardiovascular events in those with the combination of elevated TG and low HDL-C [49]. Subgroup analysis of the ACCORD Lipid study also showed benefit in type 2 diabetes patients with this atherogenic dyslipidaemia [45]. When data from the major fibrate trials were combined, individuals with atherogenic dyslipidaemia gained significant clinical benefit for reduction in risk of cardiovascular events, whereas those without this lipid profile did not [50].

Longer-term follow-up of the BIP study showed that patients with the combination of three of the five criteria of the metabolic syndrome derived significant benefit in terms of reduction in myocardial infarction [51]; added to this, 22-year follow-up also showed that elevated TG was a significant predictor of all-cause mortality [52]. There is also evidence to suggest a legacy cardiovascular benefit from fenofibrate treatment in the ACCORDION study, an observational follow-up of the ACCORD Lipid study [53].

There are, however, well-recognised safety concerns with the current fibrates. A major issue is an elevation in serum creatinine with fenofibrate [54]; although this is reversible, there are practical disadvantages in stopping and restarting treatment, as well as limitations to its use in patients with renal dysfunction. The potential for drug-drug interactions is another issue, most notably the risk of myopathy with statin coadministration, clearly demonstrated with gemfibrozil [55, 56].

### Other PPAR Agonists

PPARγ agonists are currently limited to pioglitazone, indicated as a glucose-lowering agent for the management of type 2 diabetes mellitus [57]. In addition to beneficial effects on atherogenic dyslipidaemia, pioglitazone has been shown to regress atherosclerosis and reduce cardiovascular events in this patient group [58–60]. The IRIS (Insulin Resistance Intervention after Stroke) trial demonstrated cardiovascular benefit in patients with insulin resistance but without diabetes [61], and significantly reduced the development of diabetes [62]. Safety issues in the trial included weight gain and increases in fracture risk and oedema [63].

Despite encouraging experimental findings, the clinical development of PPARβ/δ agonists in metabolic disorders has been disappointing. Seladelpar (MBX-8025) favourably impacted metabolic parameters, reducing apo B100, TG, non-HDL-C and C-reactive protein and increasing HDL-C, in a short-term trial in overweight men and women with mixed dyslipidaemia, with and without atorvastatin treatment [64]. There was, however, no benefit in NASH, leading to termination of clinical development of this agent [65].

Improved understanding of interactions at the PPAR receptor has invigorated the search for selective and potent peroxisome proliferator-activated modulators (SPPARMs), which aim to maximise beneficial effects and minimise the adverse effects of current PPAR agonists [28••].

## SPPARMs for the Metabolic Syndrome?

The underlying aim of a SPPARM is to improve specificity and potency (i.e. efficacy) and minimise safety issues with established PPAR agonists, such as fibrates. This rationale borrows from that used in the development of selective oestrogen receptor modulators [66]. Understanding binding interactions at the PPAR has been key to the development of SPPARMs, as previously discussed [28••]. In brief, binding of the ligand (drug) at the receptor induces specific conformational changes and selective recruitment of coactivators which then selectively activate or repress key target genes, with downstream therapeutic effects. This is the paradigm on which the search for a SPPARM is based.

In the history of SPPARM development, some have shown dual activity, such as aleglitazar, a PPARα/γ agonist (terminated) and elafibranor (previously known as GFT505), a PPARα/δ agonist, which had been targeted to the management of NASH. The latest interim analysis of the RESOLVE-IT Phase 3 trial with elafibranor, however, failed to show a significant benefit for the predefined primary endpoint of NASH resolution without worsening of fibrosis, or secondary endpoints related to metabolic parameters versus placebo [67]. Most recently, lanifibranor, a pan-PPAR agonist, met primary and secondary endpoints in a phase 2b study in NASH [68, 69].

The very few pure SPPARMα agonists that have been developed to date include LY518674, GW7647 and most recently, pemafibrate (previously referred to as K-877), now licensed in Japan for the management of dyslipidaemia [70]. LY518674 potently upregulated apo A-I production and catabolism in human subjects with the metabolic syndrome [71] but was not superior to fenofibrate in lowering TG and raising HDL-C in patients with atherogenic dyslipidaemia. Additionally, elevation in serum creatinine (similar to that observed with fenofibrate) was also reported with this agent [72].

**Fig. 2:**
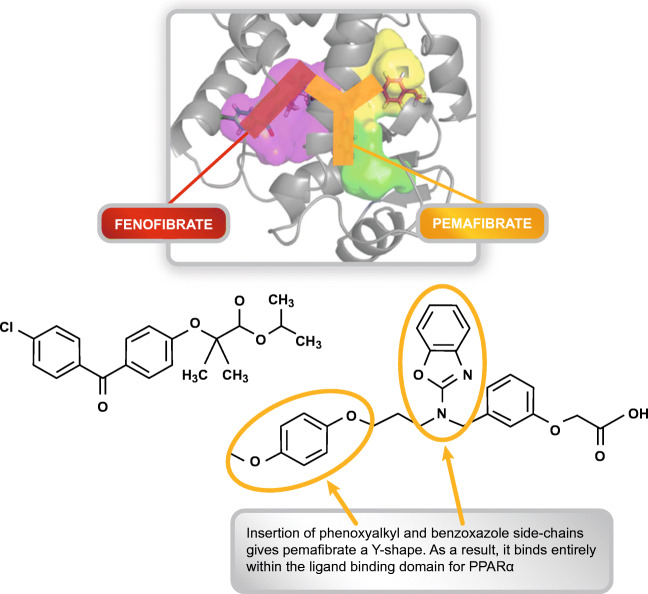
Pemafibrate is the realisation of the SPPARMα concept. Understanding binding interactions at the PPAR have been critical in driving the development of SPPARMα. Systematic structural modifications based on a precision medicine approach led to the creation of pemafibrate. This agent demonstrated an enhanced fit completely within the ligand binding domain of PPARα, in contrast to the linear structure of conventional fibrates such as fenofibrate. PPARα, peroxisome proliferator-activated receptor alpha

Pemafibrate is the culmination of the systematic synthesis of over 1500 compounds which were rigorously screened for SPPARMα activity. As for traditional PPARα agonists, the pemafibrate molecule has an acidic region, but the addition of unique benzoxazole and phenoxyalkyl sidechains gives it a Y-shaped structure. As a result, pemafibrate has an enhanced fit within the ligand-binding domain of the PPARα (Fig. 2) [73, 74, 75•]. This structural differentiation confers an increase in PPARα activation potency compared with other fibrates and a high degree of PPARα subtype selectivity [28, 76]. Transcriptome analysis showed that gene expression profiles also differed between these two agents, particularly in terms of magnitude of effect. For example, pemafibrate induced key target genes such as *VLDLR* and *ABCA1* at a concentration 10-fold lower than fenofibrate [77].

In preclinical studies, there was more robust reduction in TG and elevation in HDL-C with pemafibrate compared with fenofibrate, as well as enhanced cholesterol efflux from macrophages, upregulation of fibroblast growth factor 21 (FGF-21), reduced inflammation and attenuation of atherosclerosis [78]. Additionally, pemafibrate attenuated postprandial hypertriglyceridaemia in a mouse model [79], by suppressing the postprandial increase in chylomicrons and the accumulation of chylomicron remnants. This response was achieved with a pemafibrate dose 100-fold lower than with fenofibrate [79], implying that pemafibrate is more efficient in decreasing TG and apo B48-containing chylomicron remnants, which are highly atherogenic lipoproteins, more so than those containing apoB100 [80, 81].

Insights from rodent models of NASH have suggested that pemafibrate may have potential in NASH. In a diet-induced amylin NASH mouse model, pemafibrate improved dyslipidaemia, liver dysfunction and NASH features. These effects were attributed in part to stimulation of lipid turnover [82•]. A subsequent study using a STAM NASH mouse model which demonstrates NASH progression resembling the clinical disease showed that pemafibrate improved the histological severity of NASH, as well as inflammatory and fibrosis marker gene expression, without influencing hepatic TG content [83•]. The findings from this study are therefore consistent with current thinking that combination therapy targeting multiple components is needed to manage NASH [84].

## Pemafibrate in Metabolic Syndrome: Clinical Profiling

Preclinical data support the SPPARMα concept and suggest potential therapeutic application in managing dyslipidaemia associated with the metabolic syndrome. Additionally, because pemafibrate is metabolised in the liver and excreted into the bile [85], it can be used safely in patients with renal impairment, as borne out in clinical trials [86, 87•].

An early study in Japanese subjects with atherogenic dyslipidaemia (TG > 200 mg/dL and low HDL-C, < 50 mg/dL in men and < 55 mg/dL in women) showed robust TG-lowering (by ~ 45%) with pemafibrate (0.2 or 0.4 mg daily) that was superior to fenofibrate 100 mg daily [88]. Pemafibrate treatment was also effective against other components of the dyslipidaemia associated with the metabolic syndrome, including lowering VLDL-C (43 to 48%), remnant cholesterol (48 to 50%) and apo B and C-III, raising HDL-C (21 to 14%) and promoting a shift to a more favourable lipoprotein profile, with fewer small and very small LDL. Importantly, pemafibrate treatment was well tolerated with no increase in serum creatinine and decreased liver enzymes [88]. Pemafibrate also attenuated postprandial hyperlipidaemia [89], consistent with preclinical findings [90] and reduced inflammatory markers such as serum amyloid A and high-sensitivity C-reactive protein [89].

Furthermore, pemafibrate (0.4 mg daily for 24 weeks) was similarly effective in Japanese patients with type 2 diabetes mellitus and hypertriglyceridaemia (≥ 150 mg/dL or 1.7 mmol/L), as assessed by reduction in TG and other markers of TG-rich lipoproteins. Pemafibrate treatment also lowered fasting glucose, insulin and homeostasis model assessment of insulin resistance (HOMA-IR) levels [91]. In a hyperinsulinaemic-euglycemic clamp study in subjects with hypertriglyceridaemia and insulin resistance, pemafibrate treatment improved hepatic glucose uptake and insulin sensitivity [92]. This effect may be attributed to the stimulation of fatty acid beta-oxidation and amelioration of liver dysfunction [92] and/or mediated by the effect of increases in FGF21, as shown by this and other studies [91, 92], on insulin-dependent hepatic glucose disposal [93]. These findings imply benefit with pemafibrate beyond lipid-lowering, consistent with the pharmacology of PPARα activation.

**Fig. 3:**
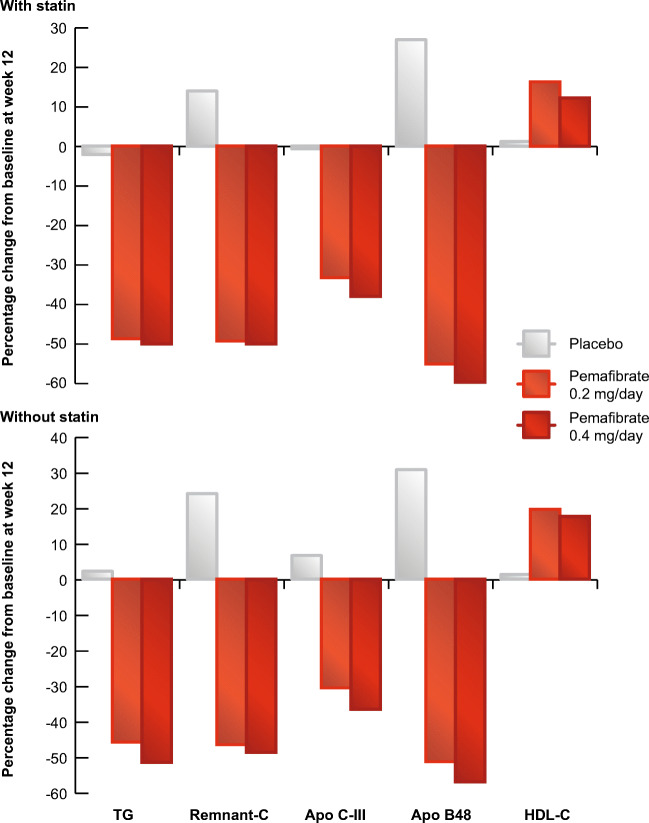
Pemafibrate favourably impacts the atherogenic dyslipidaemia of the metabolic syndrome. Pooled analysis of more than 1200 patients with atherogenic dyslipidaemia showed that pemafibrate treatment for 12 weeks lowered triglycerides (TG) and associated atherogenic lipoproteins and raised HDL-C, irrespective of statin therapy. Data from Yamashita et al [94•]. apo, apolipoprotein; C, cholesterol; HDL-C, high-density lipoprotein cholesterol

Pooled clinical trial data confirmed the favourable benefit-risk profile for pemafibrate. In one analysis including 1253 patients (677 also treated with a statin) with atherogenic dyslipidaemia in six phase II-III clinical trials [94•], pemafibrate 0.4 mg daily lowered TG by ~ 50%, irrespective of statin treatment, with almost all (98.6% on statin and 97.7% on pemafibrate monotherapy) patients showing an appropriate response. Efficacy against other components of this dyslipidaemia was robust, notably lowering remnant cholesterol by ~ 50% (Fig. 3). The safety of pemafibrate was also reassuring. Regardless of statin use, pemafibrate was well tolerated, with a favourable renal and hepatic safety profile, even among patients with mild to moderate renal impairment [86]. There was no evidence of interaction with concomitant statin therapy [94•].

Finally, the PROVIDE study provided insights regarding the long-term efficacy and safety of pemafibrate (0.2 or 0.4 mg daily for 52 weeks) in patients with type 2 diabetes mellitus and elevated TG [95•]. Robust lowering of TG and remnant cholesterol with pemafibrate (~ 50%) was sustained over this period, together with improvement in fasting insulin and HOMA-IR. Additionally, pemafibrate treatment improved liver function tests (such as alanine aminotransferase and gamma-glutamyl transferase), and was not associated with clinically meaningful increases in creatine kinase or serum creatinine, supporting the favourable safety profile of this SPPARMα.

## Conclusions

SPPARMα poses an attractive approach to managing atherogenic dyslipidaemia associated with the metabolic syndrome. Despite early disappointment with the first compounds tested, the latest candidate, pemafibrate, has shown a promising benefit-risk profile in patients with atherogenic dyslipidaemia or hypertriglyceridaemia, key features of the metabolic syndrome. In addition to robust lowering of TG and remnant lipoproteins and elevation in HDL-C, pemafibrate treatment improved insulin sensitivity and reduced inflammation. Importantly, pemafibrate was well tolerated, with no evidence of clinically meaningful elevation in serum creatinine, a concern with conventional fibrate therapy.

The outstanding question, which has so far eluded fibrates in the setting of current evidence-based treatment including statins, is whether treatment with pemafibrate significantly reduces cardiovascular events in patients with atherogenic dyslipidaemia. This is being tested in the PROMINENT study (Pemafibrate to Reduce cardiovascular OutcoMes by reducing triglycerides IN diabetic patiENTs), a cardiovascular outcomes study in 10,000 patients with type 2 diabetes mellitus and atherogenic dyslipidaemia (TG ≥ 2·3 mmol/L and < 5·6 mmol/L, and low HDL-C), in both primary and secondary prevention settings [96••]. Results are eagerly anticipated in the next 2–3 years.

Finally, despite the ongoing obesity pandemic, there are still no approved treatments for NAFLD, the hepatic manifestation of the metabolic syndrome. As the SPPARMα pemafibrate favourably impacts lipoprotein metabolism and inflammation, it may offer therapeutic potential. Promising results in experimental NASH models and evidence of benefit in lowering liver enzymes in clinical trials are encouraging [97]. On this basis, pemafibrate is being tested in an ongoing trial in NAFLD (ClinicalTrials.gov identifier NCT03350165) [98]. It should, however, be borne in mind that the multifactorial pathogenesis of NAFLD and lack of robust surrogate trial endpoints have presented obstacles to drug development in this area.

The coming 2–3 years are critical in defining whether SPPARMα will offer new approaches to managing the metabolic syndrome and, as a consequence, reducing the associated morbidity, mortality and disability of ASCVD.

## Data Availability

Data used during the current review are available from the corresponding author on reasonable request.
